# Researching Body Perception: Toward an Integration of Quantitative and Qualitative Interdisciplinary Approaches to Address the Multiplicity of Bodily Experiences

**DOI:** 10.1111/psyp.70234

**Published:** 2026-01-24

**Authors:** Marte Roel Lesur, Laia Turmo Vidal, Matthew R. Longo, Jenny Slatman, Aleksander Väljamäe, Ana Tajadura‐Jiménez

**Affiliations:** ^1^ i_mBODY Lab, DEI Interactive Systems Group, Department of Computer Science and Engineering Universidad Carlos III de Madrid Madrid Spain; ^2^ Department of Psychology University of Zurich Zurich Switzerland; ^3^ BeAnotherLab Barcelona Spain; ^4^ KTH Royal Institute of Technology Stockholm Sweden; ^5^ School of Psychological Sciences Birkbeck University of London London UK; ^6^ Department of Culture Studies Tilburg School of Humanities and Digital Sciences Tilburg the Netherlands; ^7^ University of Tartu Tartu Estonia; ^8^ UCL Interaction Centre University College London London UK

**Keywords:** body perception, body transformation experiences, design research, mixed methods, multisensory integration, perception

## Abstract

Experimental psychology and cognitive neuroscience have attempted to understand the underlying functioning of one's body experience. This has resulted in standardized methods involving multisensory manipulations and physiological, behavioral, and subjective measures. These approaches contribute to the important goal of creating a cumulative and reproducible science. However, they may sometimes lead to some well‐known pitfalls; most notably, construct ambiguity, measure dissociations, and loss of nuance. In this argumentative work, we propose that combining qualitative methods employed in design research centred on body experiences, together with quantitative approaches from psychology and cognitive neuroscience, can yield a richer account without compromising quantitative rigor. This integration of quantitative and qualitative methods, we argue, may be particularly valuable when dealing with one's body perception. Without pretending to fundamentally solve methodological discrepancies between qualitative and quantitative approaches, we propose a conciliatory take. We propose a three‐staged integration model that may be mapped to three common steps of the research inquiry: the experimental design, data collection, and data analysis. We further provide a synthesis of qualitative tools and methods to support the explicit reporting of exploratory practices that often remain informal in quantitative research. Our suggested mixed methods approach aims to account for individual differences, produce more nuanced insights, increase transparency, foster multidisciplinarity, and potentially speed progress in some aspects of the research program.

## Abstract 2

This study contributes to advancing body perception research by complementing existing psychophysiological and behavioral quantitative approaches with qualitative methods from design research. Given the uniqueness of body perception compared to other aspects of sensory experience, a mixed‐methods framework is essential to capture subtle dimensions of experience that may be overlooked by conventional protocols. We offer a pragmatic three‐step approach and a set of tools set to enhance transparency, methodological rigor, and experimental efficiency while fostering multidisciplinarity.

## 1 Introduction—Epistemologies Within Experimental Psychology and Cognitive Neuroscience of One's Body Experience

Within the fields of experimental psychology and cognitive neuroscience of body perception, researchers have attempted to understand the experience of one's body relying on inventive methods. Experimental paradigms to alter body perception through controlled multisensory stimulation are a standard tool in this field (Blanke 2012; Ehrsson 2012; Kilteni et al. 2015; Tajadura‐Jiménez et al. 2022). Though early explorations exist (Lackner 1988; Stratton 1899; Tastevin 1937), it was arguably with the advent of the Rubber Hand Illusion (RHI, Botvinick and Cohen 1998; see explanation below) that this line of work developed more energetically, now comprising a vast literature. In this process, a series of concepts (e.g., the construct of body ownership) and methodologies (e.g., measuring electrodermal activity when threatening the fake hand during the RHI; see below) have become standard. And while these might be the best tools and conceptual frameworks at our disposal for a reproducible and cumulative science of bodily experience, they might also entail overlooked limitations (Overgaard 2023; Raoul and Grosbras 2023; Timmermann et al. 2023). In what follows, rather than presenting new experimental data, we argumentatively address some of these shortcomings to uncover potential opportunities by complementing existing psychophysiological and behavioral approaches in psychology and cognitive neuroscience. We propose a concrete three‐stage mixed‐methods approach that draws upon qualitative methods from design research and seeks transparency of qualitative practices that may be informally applied in experimental research. To distinguish our approach from the significant tradition in neuropsychology, psychiatry, and neurology based on patients' reports and case studies that aim to formalize altered bodily experiences (Brugger and Lenggenhager 2014; Critchley 1950), here we focus on experimental research.

Commonly, experimental paradigms in this field rely on a manipulation of bodily cues in one sensory modality that are mapped to our experience in another modality. This creates a cross‐modal conflict that results in an altered bodily experience upon multisensory integration (Blanke et al. 2015; Botvinick and Cohen 1998; Ehrsson 2012; Kilteni et al. 2015). For instance, in the RHI, a rubber hand is placed in a plausible position within the participants' field of view and touched while their own hand is occluded and simultaneously touched (Botvinick and Cohen 1998). This may lead to the experience of ownership over the rubber hand, which is thought to result from mapping the felt touch (on the participants' own hand) to the seen touch (on the rubber hand). Similar paradigms exist across sensory modalities, including a tactile‐motor (i.e., excluding vision) version of the RHI (see Ehrsson et al. 2005; Galigani et al. 2022; Petkova et al. 2012; Radziun et al. 2023) among other ingenious setups that may result in remarkable perceptual alterations (e.g., Ley‐Flores et al. 2022 using auditory‐motor associations; Roel Lesur, Aicher, et al. 2020, visuo‐olfactory; Tajadura‐Jiménez et al. 2012, auditory‐tactile). The resulting experiences are generally referred to as bodily illusions (but see Dieguez 2018; Roel Lesur et al. 2018a).

In experimental psychology and cognitive neuroscience three main sources of quantitative observation have been used to operationalize this experience. First, *subjective* reports in the form of scales (e.g., quantifying “I felt that the rubber hand was my own hand” on a scale; Longo, Schüür et al. 2008); second, *behavioral* measures that are consistent with the altered perception (e.g., judging one's hand location as closer to the rubber hand when asked to point at one's own; Botvinick and Cohen 1998; Tsakiris and Haggard 2005); and third, the typical effect found using *psychophysiological* measures, which are physiological changes in the expected direction according to the altered perception (e.g., having an increased skin conductance response to a threat toward the rubber hand; Armel and Ramachandran 2003). Quantitative findings from an experimental condition are usually compared to a control condition (in the RHI, this could be a temporal mismatch between the touch on the rubber hand and on participants' hand), and a statistically significant difference indicates a change in bodily experience. While recently these methods have been criticized on the basis of being linked to demand characteristics and hypnotisability (Lush et al. 2020, 2021), these critiques have been sensitively addressed (Ehrsson et al. 2022; Slater and Ehrsson 2022).

In addition to the earlier approaches, psychophysical procedures employ a combination of behavioral and subjective measures in relation to discernible changes in the stimulus signal and are considered a robust method in sensory research (Ehrenstein and Ehrenstein 1999). This can be a change in the stimulus signal (e.g., delay time between touch and vision) that is either subjectively discriminated or not (e.g., in terms of delay detection or ownership; see Chancel, Ehrsson, et al. 2022; Lanfranco et al. 2023; Roel Lesur, Weijs, et al. 2021), resulting in a measure that can be further interrelated to other subjective and physiological changes (Ehrenstein and Ehrenstein 1999). However, such approaches are successful for clearly distinct qualities of experience but miss more complex experiences involving different phenomenal properties (Longo, Schüür, et al. 2008).

Beyond these quantitative efforts, qualitative methods exist (Carter and Ogden 2023; Lewis et al. 2013; Lewis and Lloyd 2010; Smith et al. 2009; Valenzuela Moguillansky et al. 2013; Van Manen 2023); yet, in psychology and cognitive neuroscience, they are often undervalued, arguably because they are not generalizable and reproducible (although this may not be the goal of qualitative research; see Section 2).

In general, attempts to categorize one's body experience have resulted in a number of concepts, terminologies, and operationalizations (see Raoul and Grosbras 2023). An example is the elusive distinction between body schema and body image, which was defined early in the literature (Head and Holmes 1911) and has been articulated not without difficulty since (de Vignemont 2010; Gallagher 2005; Raoul and Grosbras 2023; Roel Lesur et al. 2018b). However, recently the relevance of these terms and their dissociation have been questioned (see Raoul and Grosbras 2023, for an extensive critical review). While they might be relevant for some fields, their distinction and interrelation are poorly characterized, and there seems to be no consensus between fields as to how they are articulated and operationalized (Raoul and Grosbras 2023). While alternative arguably more encompassing models have been proposed (de Haan and Dijkerman 2020; Longo 2016; Raoul and Grosbras 2023; Riva 2018; Schwoebel and Coslett 2005), this early terminology and distinction remain prevalent. This example portrays how concepts might be imprinted into research according to the field's conventions. While they support the inquiry's continuation and serve a pragmatic purpose, they could ignore relevant features that lay beyond such preconceptions. To address this, a mixed qualitative‐quantitative approach may support the description of overlooked perceptual, cognitive, and affective dimensions of bodily experience.

Another concept worth examining is that of body ownership. Generally, it is referred to as the feeling that a body part or full body feels like it is part of one's own body (Blanke 2012; De Vignemont 2018). It is among the most experimentally studied attributes of body experience in psychology and cognitive neuroscience. Body ownership is usually considered a feeling which emerges before conceptual awareness (Blanke 2012; De Vignemont 2018; Gallagher 2000, 2017). However, some authors holding an arguably less popular perspective contend that it is a judgment rather than a feeling. That is, that body ownership results from cognitively judging the state of one's body according to sensations across sensory modalities, rather than a distinct feeling per se (Bermúdez 2011; Milliere 2020; c.f. De Vignemont 2018; Gallagher 2017). While it is difficult to provide conclusive evidence for either perspective (De Vignemont 2018), most empirical research has considered body ownership a feeling, providing a pragmatic heuristic for investigation. However, it is not uncommon to find works using the term *objective measures* of body ownership (Campos et al. 2018; Kalckert and Ehrsson 2014; Tosi et al. 2023), a terminology that underlies the assumption of these being direct measures of such a construct rather than aspects of one's body linked to the potentially complex experience of body ownership.

In fact, these so‐called objective measures of body ownership, which refer to quantifiable behavioral or psychophysiological qualities assumed to reflect changes in body ownership, do not always go hand in hand with subjective reports. Psychophysiologically, apparently consistent and robust early findings of decreased limb temperature accompanied by changes in body ownership during the RHI (Moseley et al. 2008; Tsakiris et al. 2011) were later not replicated despite subjective changes in body ownership (de Haan et al. 2017). Behaviourally, one of the most common measures in the RHI, the proprioceptive drift (a change in the estimated location of one's hand toward the rubber hand), correlates only weakly with subjective reports (Tosi et al. 2023) and occurs sometimes in isolation from subjective changes in body ownership (Abdulkarim and Ehrsson 2016; Holmes et al. 2006; Rohde et al. 2011). For example, Rohde et al. (2011) showed that proprioceptive drift occurred regardless of synchronous visuotactile stimulation and was rather reduced during asynchronous stimulation, suggesting that it is independent of body ownership (c.f., Abdulkarim and Ehrsson 2016). These dissociations strengthen the notion of such measures being linked to the potentially complex experience of body ownership rather than objective measures of it. In fact, despite the vast literature available on the topic, some authors contend that the feeling of disembodiment is a more ecologically common and clinically relevant experience phenomenally distinct from a lack of body ownership (de Vignemont 2011; Roel Lesur, Weijs, et al. 2020, 2021), further exemplifying how research trends might be put before ecologically more common and clinically more relevant states. Note that we do not intend to say that psychophysiological and behavioral operationalizations are not useful in this field—for indeed there are physiological changes in the brain linked to clinical alterations of body perception that are also present during bodily illusions (Blanke et al. 2015; Chancel, Iriye, et al. 2022; Ehrsson et al. 2005; Gentile et al. 2013; de Haan and Dijkerman 2020; Pamplona et al. 2022)—but that our reduction of one's bodily experience to unidimensional constructs might be simplistic and overlook important phenomena (Longo, Cardozo, et al. 2008; Tosi et al. 2023).

In contrast to other perceptual features such as the visual experience of color, one's body is simultaneously the subject and object of experience, and it is not only accessible to oneself but also to others and therefore it may be subject to more complex informational layers (e.g., body ideals and social stigmas) distinct from other aspects of perception. We contend that bringing diverse personal accounts (potentially including those of researchers, participants, patients, or other specific target populations) into the research foreground might nourish a more integrative study of one's body experience without sacrificing quantification. This does so not by limiting to, but by including complementary qualitative assessments (see Corneille and Gawronski 2024), thereby going beyond prior conceptualizations of what an experience should be. It is important to acknowledge that qualitative methods have limitations too, including the difficulty to generalize findings, the inherent bias and nudging involving both researchers and experimental contexts, and that both theory and assumptions will intrinsically play a role in interpreting the data (Bradley 1993; Kerwin and Ordaz Reynoso 2021; Morgan 2007). However, these methods might also yield more intricate descriptions that consider individual differences and that go beyond preconceptions. Overall, this should contribute to a more in‐depth interpretation of psychophysiological correlates and a more discerning account of what participants mean when being confronted with questions such as what a hand feels like, which potentially involves affective, sensory, cognitive, and contextual layers (Aniulis et al. 2022; Carey et al. 2019; Crucianelli et al. 2013; Dijkerman and Lenggenhager 2018). Our aim here is to offer qualitative tools to complement psychology and cognitive neuroscience research on one's body perception, offering support and novel paths for research, including longer‐term and in‐the‐wild research (i.e., in real life rather than in laboratory settings).

## 2 Design Research as a Methodological Opportunity for Addressing Challenges Within Psychology and Cognitive Neuroscience of One's Body Experience

We find ourselves between a reductionist but generalizable approach to studying bodily experience, and a nuanced account that might evade reproducibility. We propose that simultaneously voicing participants' perspectives and fostering a more thorough understanding of individual differences, while continuing and potentially fine‐tuning quantitative methods, might reduce the limitations of each approach. In contrast to neurophenomenology's epistemological aim of solving an explanatory gap between realist (third‐person) and idealist (first‐person) views through coherent accounts from each source (Berkovich‐Ohana et al. 2020; Varela 1996), we take a more pragmatic approach: to expand and fine‐tune cognitive neuroscience's tools to investigate body perception. Though there is an overlap in attempting to highlight the nuance and dynamism of experience (Timmermann et al. 2023), we focus on providing a set of qualitative methods, examples, and a research structure that may aid our efforts to understand body perception.

Design research on body experiences might provide relevant insight to this effort (see Auernhammer et al. 2021; Höök 2018; Redström 2017). We consider this approach as applied within the field of Human‐Computer Interaction (HCI). Although design research is used across disciplines (Redström 2017), HCI is particularly well suited for investigating body perception, as it has a long tradition of researching and designing for (technologically mediated) body experiences (Höök 2018).

In contrast to the focus of experimental psychology and cognitive neuroscience on discerning psychophysiological links and the underlying functions of perceptual processes, design research on body experiences often aims to address the complexity of a subject that is embodied, embedded, enacted, and extended, aligning with phenomenological and pragmatic stances on body perception (Auernhammer et al. 2021; Redström 2017; Shusterman 2008). It is more pragmatic in its core, seeking to support the understanding, design, and assessment of how people interact and relate with and through design constructs, favoring people's experience in the research inquiry (Koskinen et al. 2011; Rogers et al. 2011). Several of its methods are shared with or derived from qualitative psychology, sociology, and the arts, and at least two stances in terms of knowledge generalizability exist. Qualitative data can be analyzed in ways that can be extended to others beyond the sample, not intending to reproduce and extrapolate, but rather to identify themes and patterns (Braun and Clarke 2006). For example, by finding common analytical patterns within people's experience interacting with a technology (Löwgren 2009). An alternative approach, common within design research in HCI, is known as *intermediate level knowledge* (Höök and Löwgren 2012; Löwgren 2013). It stands between specific, anecdotal accounts and claims of generalizability. Its knowledge forms often consist of methods, qualities, concepts, or recommendations. This type of knowledge could be understood in experimental psychology and cognitive neuroscience as an indicator to pursue a plausible idea that might lie beyond readily existing operationalizations or without existing quantitative data in which to base new hypotheses.

Design research has been articulated as an iterative process that goes back and forth between different phases of the investigation (e.g., understanding, development, testing, analyzing, iterating, deploying) to assess and finetune tools and methodologies, and derive knowledge in the process (Auernhammer et al. 2021; Koskinen et al. 2011; Rogers et al. 2011). As we will argue in the following sections, some of these qualitative approaches are commonly used; however, informally or without being reported in detail, within experimental psychology and cognitive neuroscience (Longo, Schüür, et al. 2008; Roel Lesur, Lyn, et al. 2020; Roel Lesur, Weijs, et al. 2021).

**FIGURE 1:**
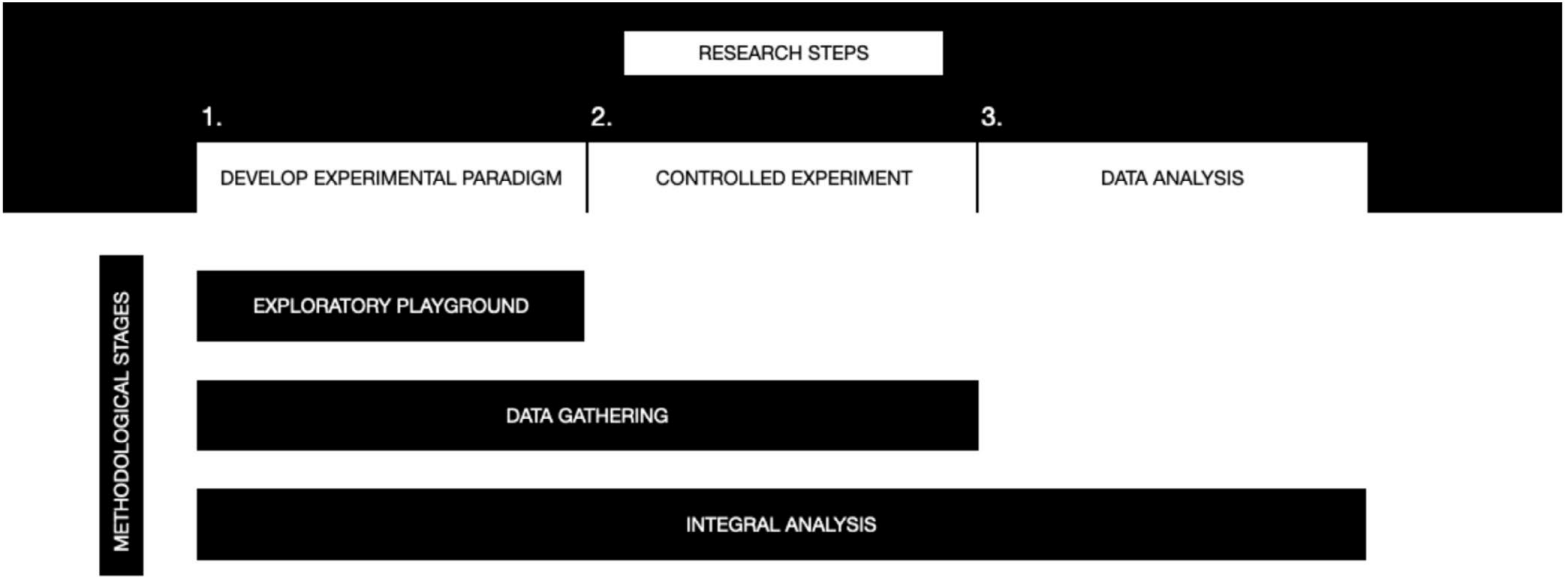
Three common research steps in experimental psychology and cognitive neuroscience research are depicted horizontally above. Below, vertically, three methodological stages for body perception research are proposed. These draw from design research and can be employed at each of the research steps above. These steps and stages are not to be taken (solely) sequentially, but they may be approached in an iterative manner. That is, insights from one step or stage can be employed to iterate other steps or stages of the research process, for example, new explorations, research questions, and tools being constructed on the basis of analytical findings.

Three common steps within psychological research on bodily experience can potentially benefit from our proposed three‐stage iterative methodology (Figure 1). First, the development of the experimental protocol and measures of interest could be supported by an “exploratory playground” as we term it. This may include qualitative and quantitative data gathering and analysis with experts and populations of interest for the specific, yet early, research goals. It can help to fine‐tune the research questions, test and enhance the apparatuses, and distil the foci of interest. Second, the controlled experiment itself could employ qualitative tools to accompany quantitative ones. This can offer insights into people's lived experiences (Corneille and Gawronski 2024) and open paths for potential confirmation and future research. And third, the integrative analysis of mixed data that may offer multifaceted understanding and knowledge outputs (e.g., related to individual differences), which might lead to further refinements and iterations of the proposed stages. While this approach might benefit other fields in psychology and cognitive neuroscience, it is aimed to aid body perception research. As mentioned in the Introduction, body perception is subject to complex informational layers in contrast to other sensory perceptual features. Therefore, quantifying it according to single perceptual dimensions might overlook significant phenomena. Addressing nuance might in turn support the development of less ambiguous constructs and aid our understanding of dissociations between measures. Having said this, it is important to state that not always including qualitative findings might prove beneficial, and it depends on the researchers' goals. We here provide a set of alternatives to conventional quantitative efforts that might serve some readers.

**FIGURE 2:**
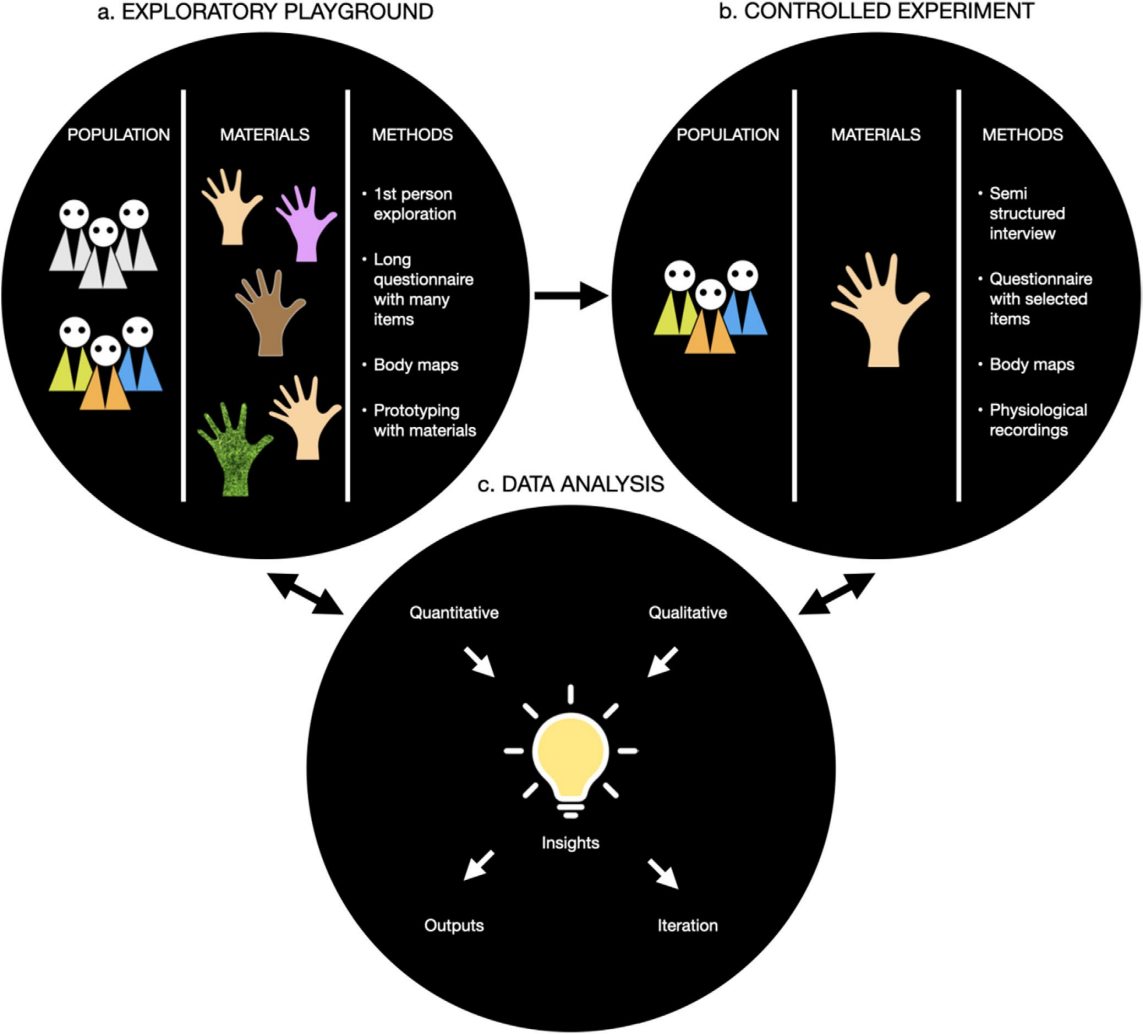
Stages of the iterative process using an imaginary example exploring the materiality of the artificial hand in relation to the RHI. (a) An explorative playground involving diverse stakeholders (e.g., naive participants—depicted in various colors to represent diverse perspectives—and researchers—in gray, though also with diverse experiences), artificial hands with different qualities and a range of methods. (b) A controlled experiment combining quantitative and qualitative methods where the insights derived from the previous step are assessed in a controlled manner with naive participants. (c) Integration of quantitative and qualitative insights for further research iterations or into research outputs. Insights from any phase may feed back to iterations of the same or other phases.

To develop our proposal across the following sections, we rely on an imaginary example about a novel study on the materiality of the artificial hand in relation to the RHI (Figure 2). In this example, first an exploratory playground is organized (Figure 2a). Here researchers and target populations engage in dialogue and first‐person explorations of various dummy hands. Different properties including hand sizes, materials, shapes, textures, and colors (Avenanti et al. 2010; Farmer et al. 2012; Guterstam et al. 2013; Schwind et al. 2017), as well as stroking materials and different stroking speeds, are explored (Crucianelli et al. 2013, 2018). To enhance the generation of bottom‐up knowledge, beyond the dummy hands brought by the experimenters, participants may design their own using other properties such as materials or forms (Hall and Poliakoff 2020) to trigger individual and collective experimentation and potentially open unforeseen explorations (Márquez Segura, Turmo Vidal, Rostami, et al. 2016; Turmo Vidal, Vega‐Cebrián, et al. 2024). Both quantitative (e.g., questionnaires; Longo, Schüür, et al. 2008, or psychophysiological recordings; Tsakiris et al. 2011) and qualitative (e.g., group conversations or interviews) methods could be employed (Roel Lesur et al. 2024). A first analytical process could yield early insights into the population, new ideas, and a testbed of methods and approaches that can be extrapolated to future iterations of this step or for the controlled experiment to follow. As a second step (Figure 2b), the controlled experiment could be organized based on, say, an insight from the exploratory playground where a hand made out of granite was perceived as heavy and less movable compared to a humanoid rubber hand. It may involve physiological, self‐report, or behavioral measures, and it can also incorporate complementary qualitative methods (see Table 1 for some examples). Lastly, in the analysis phase (Figure 2c), the gathered mixed quantitative and qualitative data are integrated. Insights from this analysis could yield research iterations, informing further exploratory playgrounds, controlled experiments, or reach knowledge outputs (e.g., journal papers or conference proceedings) that describe the iterative mixed processes either partially or at large. Each of these stages and their justification are presented in the sections that follow.

**TABLE 1:** Examples of qualitative tools and methodologies often employed in design research that can be applied in each methodological stage (see Figure 1), as well as their objectives and potential knowledge contributions.

Methodological stages	Design research methods/tools	Objectives	Knowledge contributions
Exploratory playground	First‐person explorations and methods (Höök 2018; Höök et al. 2018) Iterative design (Rogers et al. 2011) Material exploration and building prototypes (Koskinen et al. 2011; Rogers et al. 2011; Turmo Vidal, Tajadura‐Jiménez, et al. 2024) Brainstorming (Paulus and Nijstad 2019) Bodystorming (Márquez Segura, Turmo Vidal, and Rostami 2016) Art/science collaboration or residencies (Bertrand et al. 2014; Eldred 2016; Kuusk et al. 2020; Roel Lesur, Lyn, et al. 2020; Tajadura‐Jiménez et al. 2020)	Understanding target populations Identification of individual differences and similarities Optimizing techniques for measuring and stimulation of specific phenomenologies Partially avoiding the imposition of priors Pragmatic validation Articulating and specifying research agendas Research in complex settings (in‐the‐wild) and through longer periods	Intermediate‐level knowledge (Höök and Löwgren 2012; Löwgren 2013) Research programs (Redström 2017) Best practices, guidelines, Methods (Koskinen et al. 2011; Rogers et al. 2011) Concepts (Dalsgaard and Dindler 2014; Höök and Löwgren 2012) Experiential qualities (Löwgren 2009) Thick descriptions (Freeman 2014) Acknowledging complex settings and through extended time periods (Ley‐Flores et al. 2021)
Data gathering	Body maps (Turmo Vidal et al. 2023) (Semi)structured interviews, observations, focus groups (Rogers et al. 2011) Micro‐phenomenological interview and reflections (Prpa et al. 2020) Sensory ethnography (Pink 2015) Informal conversations (Swain and Spire 2020; Welsh and Wright 2010) Auto‐ethnography (Ellis and Bochner 2000; Ghita 2019) Movement‐based design methods (Vega‐Cebrián et al. 2023)		
Data analysis	Thematic analysis (Braun and Clarke 2006) Content analysis (Drisko and Maschi 2016) Integrative mixed‐data practices (Fetters et al. 2013)		

*Note:* These might help integrate qualitative approaches and expand knowledge forms in experimental psychology and cognitive neuroscience of one's body experience.

### 2.1 Exploratory Playground: Design Research Methods for Developing Experimental Paradigms

One could argue that the iterative procedures of testing, iterating, and fine‐tuning tools and methods that are common and well‐articulated within design research (Koskinen et al. 2011; Rogers et al. 2011) are also implicitly used within experimental psychology and cognitive neuroscience. In fact, experimental studies in this field often begin with first‐hand phenomenological explorations by the researchers that may turn into group discussions and iterative experimentation, where protocols, technical tools, methods, and research foci are updated to eventually become a controlled experimental paradigm (Longo, Schüür, et al. 2008; Roel Lesur, Aicher, et al. 2020; Roel Lesur, Bolt, et al. 2021; Roel Lesur et al. 2024). In this line of work, however, this part of the research is generally not mentioned in publications and may even be hidden from the reviewers' and readers' potential judgment. It is not considered formal practice, yet it is central to accomplish the endeavors that follow. In this sense, rich, mature, and well‐articulated methodologies from design research might prove important aids to justify and enrich this meaningful practice. By integrating them, the reliance on qualitative processes becomes explicit and articulated rather than obscured.

Within design research, this process regularly involves researchers but also non‐experts or other target groups, which is referred to as *user‐centred research* or *participatory design* (Rogers et al. 2011, see e.g., Ley‐Flores et al. 2021; Roel Lesur et al. 2024 for examples in the field of body perception). Our example from Figure 2a shows how from the exploratory playground with participants, some relevant phenomenologies and materials for sensory stimulation are derived for designing a controlled experiment. This first stage of the research process may optimize the research agenda, arriving at phenomena of interest more quickly—including those that might otherwise remain undiscovered—and without requiring extensive human and economic resources, before a controlled experimental validation. New paradigms, interventions, or ideas for novel psychophysiological measures that could be worthwhile exploring, but which are too costly for quantitative research may also benefit from these methods.

A useful example of an exploratory playground comes from Roel Lesur et al. (2024). In a 3‐h workshop with 12 people including cognitive scientists, experts in HCI, sound engineers, artists, and movement practitioners, the authors set out to determine auditory‐motor mappings that lead to body perception changes. In a first phase, participants tested several sonifications of movement and evaluated them according to their most salient features in terms of bodily changes. From there, the two movement sonifications for which a majority of participants experienced similar bodily changes were chosen for a more in‐depth exploration in a second phase. This involved a thorough exploration of each sound by each participant followed by the production of body maps, questionnaires, and a joint conversation that was later transcribed and analyzed. According to the body maps, one of the sonifications produced bodily sensations across the whole body, while the other primarily on the upper part of the body. The questionnaires and conversations further revealed that participants felt more powerful, boundless, and stronger in the condition affecting the whole body. This example portrays an efficient and data‐driven selection of auditory‐motor mappings leading to distinct bodily changes which can form the basis for a controlled hypothesis‐based experiment (for instance focused on perceived physical strength during physical therapy). Note that this strategy is particularly powerful for early stages of the research inquiry and might be followed by a subsequent exploratory playground focused on measures to capture the reported experience. Though the exact sample size, duration and phases of the study will depend on the specific research goals, we hope that this practical example is helpful to readers.

### 2.2 Controlled Experiments: Design Research Methods for Expansive Data Collection

Often within quantitative experimental protocols, conversations between participants and researchers occur after the experiment. Whether as formal or informal interviews, the findings from such debriefings are generally not considered beyond the researchers' own knowledge or internal lab discussions, and rarely do they reach a publication even if they might influence the path and understanding of the study (Roel Lesur, Bolt, et al. 2021). The reason for this lack of acknowledgment may be their potential irreproducibility, and that participants at such a stage may be biased and narrate their experience according to the researchers' conceptual framing. This is accentuated by a lack of clear guidelines, methodologies, and good practice examples in the field about how to integrate this valuable data into primarily quantitative research. However, clear guidelines do exist (Fetters et al. 2013; Swain and Spire 2020; Welsh and Wright 2010) and are important in design research (Merve Aktaş and Gümüş‐Çiftçi 2024) and other fields. As an example, in the context of science and technology studies, they form the core of the data (Chen and Ke 2016; Traweek 1992).

Indeed, linguistic exchange can be powerful for qualifying experiences with target populations (e.g., Ley‐Flores et al. 2021; Tajadura‐Jiménez et al. 2017 in the field of body perception) and is an important tool in psychology. However, beyond that, other efforts to account for participants' self‐reported body experience exist. Drawings, for example, are common in clinical neuropsychology (Smith 2009) and developmental psychology (Cox 1993) as a window into patients' perceptual universe. Another way in which drawings are used to articulate these experiences are body maps in which sensations are visually expressed within a body silhouette and allow for spatial quantification (Nummenmaa et al. 2014). This type of body map, however, offers certain constraints and priors such as a normative body shape that might prime and limit the potential responses. Certain trends thus propose reducing the amount of priors given to participants as they convey their experience (Turmo Vidal et al. 2023). For example, without highlighting the shape of a body and limiting the given instructions, these body maps rely on a bottom‐up creation of knowledge that largely stems from participants' own experiences before being framed by researchers' expectations, the limitations of quantifiable constructs, or even by normative depictions of human bodies.

Each of these approaches—primed‐silhouettes or bottom‐up body maps—offers advantages and limitations. While the former may offer spatially accurate sensation‐specific mappings (Hartmann et al. 2023; Nummenmaa et al. 2014), which can be mapped according to equidistant subdivisions of the depicted body (D'Adamo et al. 2024); the latter, beyond welcoming non‐normative bodies, could highlight novel and perhaps unexpected experiences, as well as point at recurrences that may be difficult to evoke verbally (e.g., dots or spikes depicting specific body parts). Again, while bottom‐up approaches can be difficult to generalize, they might offer clues into phenomena that may be otherwise overlooked. This is not to say that body maps are an ideal method to examine one's body experience, as they largely depend on participants' drawing and abstraction abilities, but they are another source of potentially nuanced data. Aspects of bottom‐up body maps may still be quantifiable, and, for example, involving machine learning methods to find regularities can potentially prove important for this endeavor (Correia et al. 2022). In our imaginary example (Figure 2), participants could draw body maps of their own hand after experiencing different RHI versions. Let's assume that of 20 participants, 11 of them used a thick line or filling when embodying a granite hand. This could hint at a specific phenomenology perhaps related to perceived weight to be further explored in workshops (a reiteration of step *a*), or lead to new measuring ideas for the controlled experiment (step *b*, after fine‐tuning the experimental procedure according to participants' own experiences; see Sections 2.2 and 2.3).

Design research on body experiences often looks for detailed qualitative accounts of participants' experiences as well as inductive analysis of data, rather than relying on an agenda or hypothesis. Somaesthetic research approaches, for example, place an emphasis on the lived experience of one's body (Höök 2018; Shusterman 2008). Such research favors subjective explorations of tools and methods such as micro‐phenomenological interviews (which emerged from neurophenomenology and attempt nuanced psychophysiological mappings; Bitbol and Petitmengin 2017; Timmermann et al. 2023), qualitative bottom‐up body mapping (Turmo Vidal et al. 2023), first‐person perspectives (Höök 2018), or diary studies (Ley‐Flores et al. 2021). It favors acknowledging the researchers as experimental subjects themselves, considering their first‐hand experience during research as a determining factor in knowledge construction. The knowledge derived has a generative purpose (e.g., to design artifacts) and is not necessarily extrapolatable to other participants. It might therefore imply a longer‐term engagement with a sample. Yet, as stated earlier, both a nuanced understanding of individual differences and a reconciliation with an intermediate‐level knowledge that has the pragmatic purpose of fine tuning the research agenda makes these approaches relevant. As we have argued, relying on intuitive practice and qualitative experience is present in quantitative body perception research more often than researchers tend to admit, and formalizing it could be academically beneficial.

To provide an applied example of how to include qualitative methods within a controlled experiment, we can follow our imaginary example of materiality during the RHI. Say that from our initial exploratory playgrounds (stage *a* of Figure 2), we designed a within‐subjects experiment for stage *b*, where we aimed to test how different materials (e.g., a humanoid rubber hand versus a hand with a granite appearance) influence perceived agility and ballistic movement. One experimental block can focus on a quantitative assessment including a threat‐evoked electrodermal measure to confirm the illusion (compared to a temporally asynchronous condition, see Section 1), followed by a ballistic reaching task measuring speed of movement. A subsequent experimental block could focus on the qualitative assessment of the experience, including an audio‐recorded semistructured interview assessing aspects of the experience and body maps.

The interview could include items such as: “can you describe how your hand felt?,” “did you notice any change in how easy or difficult it would be to move?,” “at what moment, if any, did it feel as though the seen hand was your own?,” “how did the material or texture of the hand affect your sense of control or responsiveness?,” “did anything surprise you or feel inconsistent during the illusion?,” “how did the knife threat feel?”. For the body maps, we thought it appropriate to depict the shape of a five‐finger hand where participants would be requested to freely draw in different colors aspects representative of their experience while making notes on the side of the sheet of what each color represented (Roel Lesur et al. 2024). In this sense, our approach provides a pre‐defined shape but allows participants to be expressive beyond a single variable in a bottom‐up fashion. It may be important at this data collection stage for experimenters to keep note of their reflections that could emerge from running the experiment and interviews in an organized manner. Though these reflections might inform the subsequent analysis, they should not be treated as data for the thematic analysis that follows, but could instead be reported to increase transparency (see Section 2.3). For our example, we decided to apply these qualitative measures in a repeated measures manner; however, whether to do it in this way or only once after all the conditions depends on the experimental focus. Naturally, repeating the same questions after each condition could potentially prime participants, whereas performing the qualitative assessment only once after the first block might dissolve the clarity of experience for each condition. As for how to analyze and present the resulting mixed data, we extend on the matter in Section 2.3.

An additional advantage of involving qualitative accounts is to follow participants through longer periods of time, to assess potential long‐lasting effects of the experimental procedures that may so far be unacknowledged. Due to the often necessary controlled settings in quantitative research, achieving this is very challenging and resource‐intensive. Combining, for example, quantitative physiological recordings via a smartwatch with a qualitative diary upon using a body transformation technology might yield relevant insights that may be overlooked in purely controlled quantitative settings. Longer assessment can, for example, study changes in body perception that may or may not have an impact (positive or negative) on daily life. Given that multisensory manipulations for altering body perception have led to changes in identity (Clausen et al. 2021; Tacikowski et al. 2020), memory (Bergouignan et al. 2014; Tacikowski et al. 2020), or agency (Kalckert and Ehrsson 2012; Kokkinara et al. 2016; Newport and Preston 2010), among others, which arguably are fundamental to wellbeing, long‐lasting effects could be important to consider as they might interfere with people's wellbeing (Yuste et al. 2017).

### 2.3 Data Analysis: Integrating Qualitative and Quantitative Accounts

Within experimental psychology and cognitive neuroscience there have been many attempts to combine quantitative and qualitative accounts (Roel Lesur, Bolt, et al. 2021; Roel Lesur et al. 2024; Timmermann et al. 2019). For example, Timmermann et al. (2019) combined several statements referring to subjective experience and their intensity on a visual analogue scale across time with electroencephalography recordings. This way, physiological signals could be linked to participants' experiences. Additionally, micro‐phenomenological interviews were performed. These interviews aim to produce awareness and articulation of one's subjective experience while reducing subjective biases and linking experiences to their neuropsychological counterpart (Timmermann et al. 2019; Petitmengin 2006). While this combination of methods is valuable, attempts to utilize it often fall short in elaborating qualitative accounts in publications (Timmermann et al. 2019). An example in the field of body perception integrating both types of data is that of Singh et al. (2016), who involved qualitative methods for an initial exploration, then continued with a more fine‐grained mixed quantitative and qualitative approach of participants in controlled settings and in‐the‐wild. This allowed for a more elaborate understanding of how movement sonifications affected body perception and attitudes toward physical activity in people with chronic pain. Yet, because it was published in the field of HCI, it might have gone unnoticed by other communities such as cognitive neuroscience and psychology (see also, e.g., Shimizu et al. 2023). In fact, it is difficult to find the appropriate journal for mixed methods works within related fields, or its within arguably smaller venues (Roel Lesur et al. 2024). In our own research, we have previously needed separate submissions for qualitative and quantitative works from the same research program on sensory illusions (Ley‐Flores et al. 2021, 2022, respectively). We make a point on the importance of welcoming such types of mixed methods data within the realm of experimental psychology and cognitive neuroscience. In the example of Figure 2, this would be combining a detailed description of stages *a*, *b*, and *c*.

The integrative analysis and reporting of such mixed methods (Figure 2c) is an existing challenge (Denzin 2009), but some guidelines suggest integration through narrative, data transformation, and joint displays (Fetters et al. 2013). The qualitative methods from Table 1 can be jointly presented with quantitative reports. Only in presenting and discussing findings, processes and novel integrative approaches might better guidelines and practices emerge, even if in some cases separate publications for each type of data could be appropriate. Novel artificial intelligence approaches may further support psychophysiological links by finding recurrences in qualitative data that link to physiological and/or behavioral measures (de Gelder and Poyo Solanas 2021). A mixed methods integration might foster more multidisciplinary exchange. Groups focusing on design or qualitative methods could find important recurring phenomena that could be worth studying in collaboration with a neuroscience or physiology lab or vice versa. Alternatively, a cognitive neuroscience group may involve design research experts in creating workshops addressing a paradigm of interest or might be trained in participatory methodologies stemming from other fields. This approach may bridge a gap between experimental psychology, cognitive neuroscience, and qualitative fields researching one's body experience, such as social sciences, HCI, design research, and even the arts. The presented approaches and the list from Table 1 will hopefully offer inspiration, but the practical ways to implement them will depend on the concrete research goals. Below, we extend on our example from Section 2.2 to address the integration of mixed data.

Following our imaginary study involving synchrony (synchronous or asynchronous stimulation) and materiality (a granite or a humanoid hand) in a RHI‐like context, let's briefly examine how we could analyze and present the findings. In our experiment, we had an experimental block focused on quantitative electrodermal responses to threat and speed of movement, and a qualitative block recording an interview and body maps. The findings from both types of qualitative results can then be subject to a reflexive thematic analysis (Braun and Clarke 2006). This analysis would follow the six‐phase methodology proposed by Braun and Clarke (2006) which consists of familiarization with the data, generating initial codes, searching for themes, reviewing themes, defining and naming them, and producing the report. A common pitfall in this approach is precisely to pretend a positivist inferential approach (perhaps due to an apparent hierarchy of this type of knowledge) rather than succinctly acknowledging the expectations, theoretical assumptions, and reasoning underlying the thematic conceptualization (see Braun and Clarke 2024a, 2024b; Carr and Lewis 2025 for discussions on good practices). In this sense, again, transparency is crucial.

Let us say that the imaginary results suggested *feeling heavier* in the synchronous granite condition was an emergent theme that followed the researchers' expectations, together with an unexpected theme of *decreased temperature*. The body maps replicated the first theme according to the notes taken, which were further expressed by thick lines or filling of the hand body map in contrast to other conditions (according to the thematic analysis). According to some guidelines, to integrate the qualitative and quantitative findings, it might prove fruitful to have a decision‐rule strategy beforehand to avoid arbitrary choices that could lead to fallacies or hinder replicability (Fetters et al. 2013), but it depends on the nature of the study. As we have argued, exploratory research should have a place when studying body perception. An example of a priori decision rules could be that, if consistency was to be found between a theme and a physiological index (e.g., reduced speed in the ballistic movement), we might retain the theme as a candidate construct for developing a questionnaire. We may then also decide to transform the qualitative data related to an emerging theme to numbers (see Fetters et al. 2013 for examples on data transformations) to perform exploratory correlations of regressions with the physiological or behavioral indexes. Also, rules for alternative scenarios could be planned. For instance, in the case that unexpected themes come up (e.g., the change in perceived temperature) or if the expected findings occur exclusively in the subjective reports.

One way to present this mixed data is a joint display, a table, matrix, or figure that aligns qualitative and quantitative results to depict how they converge, diverge or complement each other (Fetters et al. 2013). Table 2 is an example resulting from our imaginary experiment (see Fetters et al. 2013 for an example using a boxplot with data together with annotated quotes).

**TABLE 2:** An example joint display for the imaginary findings from our example study involving a humanoid and a granite hand in a RHI‐like setting.

Theme	Quantitative variable	Example qualitative quote	Participants reporting on theme	Relation between quantitative and qualitative measures
Feeling heavier	Differences between granite and humanoid synchronous conditions (*p* < 0.05 according to post hoc comparisons)	“It felt as if gravity was pulling my hand toward the surface. My hand felt really heavy”	22/30 for interviews 26/30 for body maps	Statistical differences in speed between hand conditions are in line with the reported experience (in both body maps and the interviews)
Feeling decreased temperature	NA	“It felt strangely cold, inanimate”	19/30 in the interviews 13/30 in body maps	The applied quantitative measures were not designed to capture this experience
Feeling threatened	An order effect was found (*p* < 0.01) whereas no effect or interaction for synchrony or hand conditions	“… at this stage, when you pulled out the knife, I was already expecting it, so I didn't feel threatened”	28/30 in the interviews	The applied quantitative measures seemed to reach a ceiling due to our experimental design

*Note:* We do not depict any numerical relations between the qualitative and quantitative findings in this example, but data transformations could be applied to the qualitative findings to quantify relations between variables (see Fetters et al. 2013).

Finally, let us say that according to our pre‐defined decision rule, we decided to create a novel questionnaire based on our findings. Acknowledging that this is not the place for a thorough discussion on developing psychometric scales (see Clark and Watson 1995; Hinkin 1998 for widely used examples), we provide a simplistic overview. Before creating the items it is essential to ensure clear conceptualisation of the underlying constructs, so that the eventual items offer discriminance to other related constructs (e.g., *heaviness* and *resistance to move* may be different, yet related themes), unidimensionality (i.e., the assessment of a single variable), and convergent validity (i.e., correlation with other measures of the same construct or items of the same subscale). For this purpose, it might be useful to be overinclusive at first with the questionnaire items and then reduce the set of items according to factor analyses that ideally can be later validated across independent samples. A widely used and good example in the context of body perception is the embodiment questionnaire developed by Longo, Schüür, et al. (2008).

## 3 Opportunities and Further Considerations

In this work, we adopt a pragmatic stance: to conduct research that may yield reproducible instances while voicing diverse experiences regarding one's body experience. Research in experimental psychology and cognitive neuroscience might impact participants' own opinions regarding their bodies, potentially constraining their experience and research findings. This mixed methods integration can potentially foster more multi‐ and inter‐disciplinary exchange which will hopefully lead to best practices and novel insights. While not a final epistemological resolution, we hope to expand the methodological palette for those working in experimental fields to study bodily experience. Furthermore, we open a conversation regarding the limitations of predominant methodologies for studying body perception that are often unvoiced. In the following paragraph, we outline specific opportunities emerging from this perspective, summarizing the contexts in which it may be of aid.

### 3.1 Opportunities in Fundamental Research

Some readers working in experimental psychology, psychophysiology, cognitive neuroscience, and related interdisciplinary contexts could find the proposed methods familiar. For them, given that this qualitative aspect of the research is regularly not communicated but done informally in experimental psychology and cognitive neuroscience, this work might give ground for formalization and reporting. It is also a call for addressing knowledge that is currently being lost for others. For readers new to the field or engaged in more traditional quantitative experimental fields, this work might provide useful or novel approaches and references to enrich their practice especially when it comes to new procedures. For people from qualitative fields and design research studying body perception, they may find that some of their research could be deepened by employing quantitative methods or collaborating with other behavioral and psychophysiological experts. In general, hopefully this perspective will call upon a more multidisciplinary engagement. For example, conferences on body perception could welcome more diverse perspectives, fostering a dialogue that could be beneficial to enrich the field, improve methods, and eventually articulate best practices.

Beyond this, the ongoing replication crisis in some areas of psychology and neuroscience generally demands more robust quantitative methods (Malich and Munafò 2022). Replicability issues are not rare in the study of bodily experience (de Haan et al. 2017; Mottelson et al. 2023) and would potentially benefit from more psychophysical approaches (Chancel, Ehrsson, et al. 2022; Lanfranco et al. 2023). However, as stated earlier, psychophysics and psychophysiology require simplistic dimensions of qualia, and one of the potential causes for the lack of replicability may be the complexity of the phenomena being operationalized. Our proposal takes a different approach that might contribute to the quest for solutions. By furthering our nuanced understanding of how certain things are experienced by participants, and uncovering individual differences, this in turn may shed light on why certain findings are not replicable. By no means do we intend to undermine the importance of replication, quantification and statistical insights, nor ignore issues including replicability in qualitative design research (Collins et al. 2004; Forlizzi et al. 2019). However, instead of tacitly using resources for internal reflection that are hidden from publications, relevant knowledge potentially explaining some aspects of the lack of replicability may emerge and become public.

Finally, while the need for controlled settings within quantitative approaches makes longer‐term and in‐the‐wild research difficult and costly to pursue, involving qualitative methodologies such as diaries might make investigating body experience in such contexts more accessible, at least as an initial step (see Turmo Vidal et al. 2025 for an example involving body perception). Studying how one's body experience changes through prolonged periods of sensory stimulation (Banakou et al. 2016), through the lifespan (Weijs et al. 2022), or in the wild (Roel Lesur, Lyn, et al. 2020) is elemental to further our scientific understanding of the ever‐dynamic experience of one's body.

### 3.2 Opportunities in Applied Fields

This approach could support the translation of fundamental research from different disciplines into applied fields where it might be currently unseen. In the case of HCI, it can potentially call for further multidisciplinary exchange and provide a theoretical ground for some of the work currently being done. For instance, many innovations and designs to transform one's body experience from HCI that primarily involve qualitative methods (Höök 2018) might expand their reach by involving methods from cognitive neuroscience, for instance allowing them to probe questions of generalizability. Clinically, lab‐to‐bench translations could substantially rely on our proposed approach of bridging qualitative and quantitative research, both to support expanding the clinical terminology and articulating degrees of disturbances that may aid future diagnostic criteria, and to improve therapeutic approaches and applications through customizations targeting patients' particular needs. Perhaps especially in the early stages of a novel clinical intervention, exploratory playgrounds (stage 2 of Figure 1) could optimize the intervention more efficiently and articulate what aspects of the intervention may need to be personalized, which could yield relevant case trial series. This could lead to a clinical trial mixing quantitative and qualitative methods (stages 2 and 3, Figure 2).

### 3.3 Limitations

We want to acknowledge that this perspective is not final but rather hopes to produce a necessary conversation in psychophysiological and behavioral approaches to understand one's body experience. Ideally, better methods and best practices will emerge through collaboration and acknowledgement of mixed methods. As stated, we do not mean to undermine quantitative methods, but to acknowledge elements that can be at least partially overcome by qualitative tools. We are aware of limitations specific to participants' qualitative reports, including the trustworthiness and interpretation (Bradley 1993) and its potential incommensurability with quantitative research (Denzin 2009). However, we call for the consideration of mixed methods specifically when dealing with one's body experience given its unique qualities, while recognizing that not all experimental approaches would benefit from this. For example, in psychophysics, the proposed methods could be more difficult to integrate or might even hinder the workflow due to the difficulty of directly experiencing and articulating the subtle changes accounted for by the experimental design. Similarly, processes highly susceptible to demand characteristics might not benefit from this added layer.

## 4 Conclusion

We have enunciated some issues within experimental psychology and cognitive neuroscience of one's body experience that are oftentimes unvoiced. In proposing the pragmatic use of design research that attributes significant epistemological value to participants' own rich accounts together with quantitative methods from cognitive neuroscience, we hope that some of these matters might be at least partially overcome. We have detailed a roadmap and provided an example for this type of mixed‐methods research (Figure 1) that considers formalizing common yet informal practices in experimental fields. The integration of quantitative, generalizable knowledge and nuanced accounts should be richer than separate endeavors for each of these and potentially bridge experimental psychology and cognitive neuroscience with contemporary stances in other fields regarding bodily experience. A more collective inter‐ and multidisciplinary engagement might bring forth better practices.

Looking ahead, laboratories, researchers, or new PhD students planning to study novel transformations of bodily experience according to their research parameters can potentially involve all or some of our three proposed mixed‐methods stages. First, they could begin by refining experimental manipulations through open qualitative exploration, allowing participants' lived accounts to inform and sharpen hypotheses before measurement. Next, these insights can be translated into targeted psychophysical or physiological indices for a controlled experiment, while keeping qualitative measures in the foreground. Finally, qualitative and quantitative findings should be brought back together through structured integration to identify where experience and physiology converge, diverge, or mutually inform one another. This effort could support transparency and the eventual emergence of better practices.

In conclusion, only by considering quantitative and experimental approaches, we might be able to reach generalizable insights that build a reproducible and cumulative science of body experience. And only by considering the body experience as living, dynamic, complex, embedded, and vulnerable, we might be able to reach a fair account of its nuance, variability, and richness.

## Author Contributions

**Marte Roel Lesur:** conceptualization, investigation, writing – original draft, methodology, visualization, writing – review and editing, project administration, supervision. **Laia Turmo Vidal:** conceptualization, investigation, writing – original draft, visualization, writing – review and editing, methodology. **Matthew R. Longo:** conceptualization, writing – review and editing, methodology. **Jenny Slatman:** methodology, writing – review and editing. **Aleksander Väljamäe:** conceptualization, writing – review and editing. **Ana Tajadura‐Jiménez:** conceptualization, writing – review and editing, supervision, resources, project administration, funding acquisition.

## Funding

This research was supported by the European Research Council (ERC) under the European Union's Horizon 2020 research and innovation programme (Grant 101002711) and the Dutch Research Council (NWO): VICI (Grant 277.20.008/2737). Funding for article processing costs: Universidad Carlos III de Madrid (Agreement CRUE‐Madroño 2026).

## Conflicts of Interest

The authors declare no conflicts of interest.

## Data Availability

Data sharing not applicable to this article as no datasets were generated or analyzed during the current study.
